# Relationships, love and sexuality: what the Filipino teens think and feel

**DOI:** 10.1186/1471-2458-9-282

**Published:** 2009-08-05

**Authors:** Jokin de Irala, Alfonso Osorio, Cristina López del Burgo, Vina A Belen, Filipinas O de Guzman, María del Carmen Calatrava, Antonio N Torralba

**Affiliations:** 1 Preventive Medicine and Public Health, School of Medicine, University of Navarra, Irunlarrea 1, 31008 Pamplona, Spain; 2 Department of Education, University of Navarra, 31008 Pamplona, Spain; 3 University of Asia and the Pacific, Pearl Drive cor St J Escriva Drive, Ortigas Center, Pasig City, Philippines; 4 Research for Education Intervention and Development, CRC Foundation Incorporated, Manila, Philippines; 5 Unit 1103, Pacific Center Building, San Miguel Avenue, Ortigas Center, Pasig City 1605, Philippines

## Abstract

**Background:**

In order to achieve a change among teens' sexual behavior, an important step is to improve our knowledge about their opinions concerning relationships, love and sexuality.

**Methods:**

A questionnaire including topics on relationships, love and sexuality was distributed to a target population of 4,000 Filipino students from third year high school to third year college. Participants were obtained through multi-stage sampling of clusters of universities and schools. This paper concentrates on teens aged 13 to 18.

**Results:**

Students reported that they obtained information about love and sexuality mainly from friends. However, they valued parents' opinion more than friends'. They revealed few conversations with their parents on these topics. A majority of them would like to have more information, mainly about emotion-related topics. Almost half of respondents were not aware that condoms are not 100% effective in preventing STIs or pregnancies. More girls, compared to boys, were sensitive and opposed to several types of sexism. After adjusting for sex, age and institution, the belief of 100% condom effectiveness and the approval of pornography and sexism were associated with being sexually experienced.

**Conclusion:**

There is room for further encouraging parents to talk more with their children about sexuality, specially aspects related to feelings and emotions in order to help them make better sexual choices. Indeed, teens wish to better communicate with their parents on these issues. Condoms are regarded as safer than what they really are by almost half of the participants of this study, and such incorrect knowledge seems to be associated with sexual initiation.

## Background

It is well known that, from the standpoint of public health, sexual relations among teens represent a risk factor [1-4]. Existing literature points to the alarming consequences of premature sexual involvement among adolescents [5,6]. Examining cross-country data, Wellings et al. establish that men and women in most nations begin sexual activity at ages 15 to 19 [7]. Far from settling with a marital or cohabiting partner, teens engaging in premature sex increase their risk of exposure to sexually transmitted infections (STIs) and teenage pregnancy. According to UNAIDS and the World Health Organization, the global AIDS epidemic continues to grow and the number of deaths due to AIDS is increasing in most continents [8]. Every year, 14 million adolescents give birth, which in developing countries translates to one in three women under 20 years of age [9]. Owing to the health consequences, adolescent sexual behavior is certainly a growing concern.

Competent authorities are trying to find solutions to this problem (in the form of education programs and information campaigns). However, the average age of first sexual relation is still too low, while unplanned pregnancies and STIs remain high [10-13]. Some behavioral factors such as starting sex relations at a young age and having multiple (concurrent or serial) sexual partners, increase the risk of infections [5,6,14-18]. Moreover, the use of contraceptive methods does not seem to be effective enough to avoid unplanned pregnancies in youth [19-22].

In addition to the physical dangers, existing literature has likewise examined, albeit on a smaller scale, how early sexual activity could be compromising teens' emotional and psychological well-being:

- Some studies assert that sexual activity is directly correlated to emotional problems among American teens; sexually active teenagers are more likely to be depressed and more likely to attempt suicide than teenagers who are not sexually active (even after controlling for sex, race, age and socio-economic status) [23,24].

- Personal testimonies of young people reveal that emotional dangers of premature sexual involvement are real [25].

- Most sexually experienced teens are already reporting feelings of regret over premature sexual intercourse [26,27].

Research points to different factors affecting early sex among teens. Several studies have confirmed more risky behaviors in males compared to females (higher prevalence of premarital sex, less likelihood to be sexually abstinent, increased odds of engaging in risky sex and younger age at first sexual relationship) [28-31].

Socio-economic status is also an important factor. Singh et al. ascertain that adolescent childbearing is more likely among women with low levels of income and education [32].

Several family variables have proven to be related to sexual behavior. Parent-child communication is protective against early sex [30,33,34], especially for girls [33]. Furthermore, according to the systematic review of American youth studies done by Buhi and Goodson, the youth's perception of parental attitudes toward sex is a stable predictor of sexual behavior outcomes [35].

Several studies show that the sources of information available to teens as regards sexuality are incomplete and inappropriate. A study in Costa Rica concludes that a more complete biological information is received compared to affective information. Furthermore, the same study reports that educational institutions are the most frequently used source, while the family stands in second place [36]. A Spanish research calls attention to the fact that almost half of the youth between ages 18 and 29 describe communication with their parents on sexual matters as inexistent (25.9%) and unsatisfactory (20.6%). While parents are the youth's favorite source of information, the youth in actuality turn to friends or partners for information [37].

Limiting current perspectives to the physical or biological dimensions of sexuality may further obscure fitting solutions. If intervention programs and future research are to be responsive to the needs of teens, what they feel and say should have weight in ongoing discussions. Expanding this research area has therefore the potential of uncovering important and useful insights on how to best help teens.

This research is the first step toward an international study (Project YOUR LIFE), on what the youth think and feel about relationships, love and sexuality; with the general objective of enabling future health education programs focusing on character and sex education to be grounded on youth's opinions and needs.

In particular, this paper seeks:

1. To know which is the preferred and actual main source of information about relationships, love and sexuality on representative samples of Filipino teen students;

2. To explore what topics the teens would want to know more about; and

3. To study their actual knowledge about the prevention of STIs and unplanned pregnancies as well as their attitudes toward specific issues such as sexism.

## Methods

### Data Instrument

In order to accomplish the research objectives, a paper-pencil questionnaire was crafted to gather data on the following categories: Socio-demographic characteristics; characteristics of the group of friends; use of free time; access and exposure to media; feelings, opinions and information sources on relationships, love and sexuality; and life goals.

The instrument consisted mainly of close-ended questions. A five-point Likert scale was used for attitudinal responses. The questionnaire was drafted in colloquial English and pre-tested in the field to 180 students. Questions were tested to ensure clarity, comprehension and suitability to local conditions. Content and length of the instrument was modified to last about 45 minutes.

Specifically, variables considered in this article refer to: youth's sources of information about love and sexuality; importance of parents' and friends' opinion about different topics; frequency of conversations with parents about different topics regarding sexuality, and desire to know more about these topics; degree of agreement with sentences showing disapproval towards different forms of sexism; knowledge about condom effectiveness; and sexual experience (whether the subject has had any sexual relation).

The wording of the questions and answer scales is described below where appropriate. The questionnaire is available upon request to the corresponding author.

### The sample

The targeted study population was 4,000 students from third year high school to third year college in the Philippines. Subjects were obtained through multi-stage sampling of clusters of universities and schools.

Time and budget constraints yielded the limitation of choosing seven respondent regions out of the seventeen political regions. These are National Capital Region, CALABARZON, Central Luzon, Western Visayas, Central Visayas, Davao and Northern Mindanao. The respondent regions were selected on the basis of having the greatest number of youth population while limiting two regions each from Luzon, Visayas and Mindanao (the three island groups), plus the National Capital Region.

**Figure 1:**
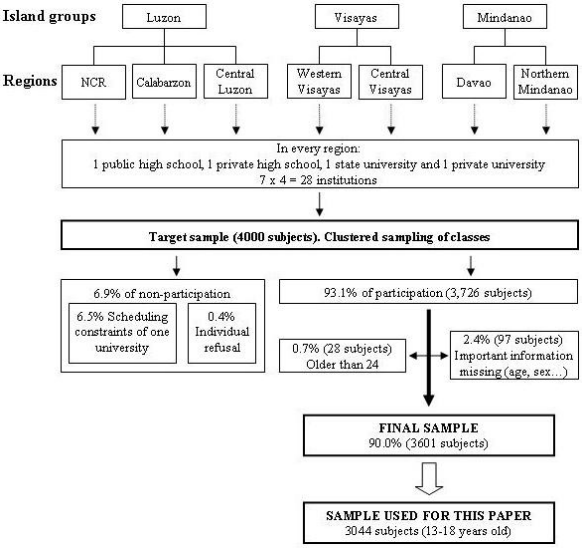
**Sampling process**.

From each region, four institutions were identified as survey venues: one public high school, one state college or university, one private high school and one private university. Schools with wider representation of youth sectors (judgment-based) were chosen (Figure 1).

The total of approximately 4000 students were targeted from the seven regions based on the respective contribution of the region to the total youth population. This sample size was chosen taking into account approximate sample size estimation criteria [38,39]. We worked with the criteria that 10 subjects would be needed per parameter included in a statistical model used to adjust for confounding. By parameter we mean each continuous variable and/or each dummy variable from categorical variables, that could be included in a model. Thus with a sample of about 4000 students we were quite confident to have sufficient statistical power to account for a good amount of variables in a given model. Equal samples were taken from each year level and from public and private sectors to improve subgroup analyses by school type. Classes were randomly selected.

Not included in the population were out-of-school youth. Priority was given to study in-school youth since one of the implicit objectives of the research is to generate insights on future formation channels for this specific group.

Finally, for the propose of the analyses of this paper, we focused only on high school students aged 13–18.

### Data Collection

The questionnaire was implemented between July and September 2007 in twenty-eight schools from seven regions using standardized data-collection protocols. Prior to administering the survey to students, consent was obtained through the schools. Schools were invited to voluntarily participate in the research project, which was described to the schools as an effort to collect nationwide baseline data to guide future education interventions.

Data collectors travelled to each participating school to administer the survey sheets during class hours. Administration in schools (that is away from parents) has the reported benefit of increasing the respondents' sense of privacy and their willingness to disclose sensitive information.

Survey procedures were designed to protect student privacy by allowing for anonymous participation. Data collectors read a standardized script, including an introduction to the survey requesting the participation of students. The survey's scope and respondent anonymity with respect to the school and their parents was explained. Moreover, students were instructed that they might opt to leave any discomforting survey item blank. The survey was completed in approximately 45 minutes or one class period in classrooms or lecture halls. To the extent possible, students' desks were spread throughout the classroom to minimize the chance that students' could see each other's responses. Neither the survey administrators nor classroom teachers moved around the classroom while students took the survey. Students were told of the importance of providing honest answers and that no one would know how they responded individually. When students completed their survey sheet, they were asked to seal their answers in individual envelopes to be returned to data collectors. Lead researchers secured and transported survey sheets to Manila for data entry.

Analysis was jointly conducted at the University of Asia and the Pacific, Philippines and at the University of Navarra, Spain. Ethical authorization was obtained for the study by the Ethics Committee of the University of Asia and the Pacific.

### Analysis

Data were analyzed taking the weights and clusters of the sampling process into account by using specific survey commands of the STATA statistical package release 9. The survey mean. proportion and logistic commands of STATA enable the estimation of group means, proportions and logistic regression respectively assuming weights and cluster sampling and thus estimating appropriate estimates and standard errors. Significance levels of comparisons and model coefficients are performed by STATA survey commands using an Adjusted Wald test [40].

## Results

The survey was answered by 3,726 subjects (93% of the targeted population). Most of the 7% of non-participation (6.5%) was due to scheduling constraints of one institution. Responses of 28 students were omitted because they were older than the target sample age (13 to 24 years). Seventy-three subjects did not give age information, 2 did not give sex information and 22 did not specify whether their school or university was public or private. Therefore, 3601 respondents were used for the project (90.0% of the targeted population). Among these, 3044 subjects (high school students, 13–18) were analyzed in this paper.

A majority of the respondents were female (64.3%) between 16 to 18 years old (60.4%). Most of them were Roman Catholics (83.6%) and came from middle-income families (79.1%) and public schools (54.3%) (Table 1).

**Table 1:** Distribution of Respondents by Key Demographic Characteristics

Characteristics	Total (N = 3044)	Male (N = 1088)	**Female **(N = 1956)
	n (%)	n (%)	n (%)
**Sex**			
Male	1088 (35.7)		
Female	1956 (64.3)		
TOTAL	3044 (100.0)		

**Age**			
13–15	1204 (39.6)	449 (41.3)	755 (38.6)
16–18	1840 (60.4)	639 (58.7)	1201 (61.4)
TOTAL	3044 (100.0)	1088 (100.0)	1956 (100.0)

**Religion**			
No religion	54 (1.8)	28 (2.6)	26 (1.3)
Catholic	2546 (83.6)	914 (84.0)	1632 (83.4)
Protestant	299 (9.8)	103 (9.5)	196 (10.0)
Others *	145 (4.8)	43 (4.0)	102 (5.2)
TOTAL	3044 (100.0)	1088 (100.0)	1956 (100.0)

**Economic status^†^**			
Low	391 (12.8)	155 (14.2)	236 (12.1)
Middle	2407 (79.1)	858 (78.9)	1549 (79.2)
High	246 (8.1)	75 (6.9)	171 (8.7)
TOTAL	3044 (100.0)	1088 (100.0)	1956 (100.0)

**School**			
Public	1652 (54.3)	598 (55.0)	1054 (53.9)
Private	1392 (45.7)	490 (45.0)	902 (46.1)
TOTAL	3044 (100.0)	1088 (100.0)	1956 (100.0)

* Other religions include Jewish, Muslim, Hindu, Buddhist, INK (Iglesia ni Kristo), Aglipay, 7th day, Pentecost, Mormon, Jehova's, Baptist, Filipinist and "other".

^† ^Based on the respondents' perception of family economic status.

Participants were asked how often they got information regarding love and sexuality from different sources. The source most often marked as "always" or "almost always" by males and females respectively, was, by far, friends (57.5% and 69.6%), followed, in the case of males, by the Internet and youth magazines (27.1%); and, in the case of females, by parents (30.7%) (data not shown).

**Figure 2:**
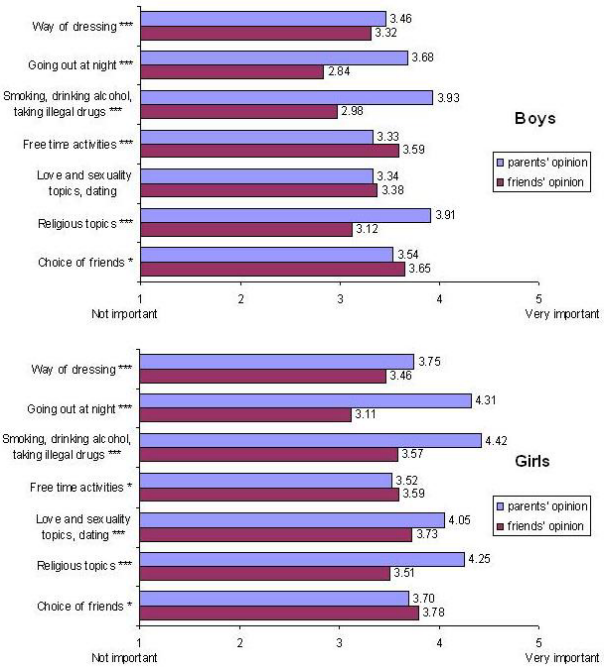
**Teens' reported level of importance of parents' vs. friends' opinion by areas of concern**. Values are the average scores obtained in each item (in a five-point Likert scale labeled from a low "Not important" to a high "Vey important" score). p value of the adjusted Wald test taking into account the clustered sampling scheme: * p < 0.05. *** p < 0.001.

The questionnaire examined how parents' and friends' opinions regarding love, sexuality and other related topics were valued by the youth. Generally, it is observed that the youth (specially girls) value parents' opinion more than friends' in most topics (Figure 2).

Parents' and friends' opinions are better appreciated by girls (compared to boys) in all topics. This difference is statistically significant for parents' opinion (p < 0.003 in "choice of friends" and p < 0.001 in all the other topics), and for friends' opinion except for "free time activities" (p = 0.005 for "way of dressing", p = 0.011 for "choice of friends" and p < 0.001 in the other topics) (data not shown).

When asked whether they have talked with their parents about the different aspects of sexuality (biological as well as affective/emotional aspects), they reported relatively few conversations with their parents. Concerning biological aspects of sexuality, topics mostly discussed with parents were, for males, pregnancy (21.7%) and STIs (20.5%); and for females, girls' physical changes (58.9%) and pregnancy (41.1%). On topics regarding feelings and relations, respondents mostly talked about how to better manage feelings and emotions (32.7% for boys, 44.8% for girls), and how to know if the person they are dating is the right one (26.4% and 36.7%) (Table 2).

**Table 2:** Conversations with parents and desire to know more

TOPICS	Talked to parents "quite a lot" or "a lot" *			Want to know more^†^		
		
	Males	Females		Males	Females	
	n (%)^‡^	n (%)^‡^	p^§^	n (%)^‡^	n (%)^‡^	p^§^
**Topics related to Biology**						
Girls' physical changes (menstruation, breast ...)	90 (9.0)	1161 (59.9)	<0.001	423 (44.0)	1454 (81.1)	<0.001
Boys' physical changes (beard, wet dreams ...)	204 (18.8)	174 (10.7)	<0.001	695 (67.9)	759 (47.5)	<0.001
AIDS and other sexually transmitted infections	235 (21.7)	491 (25.7)	0.026	811 (78.7)	1496 (83.3)	0.007
Pregnancy, the beginning of life	251 (23.9)	800 (41.7)	<0.001	672 (66.8)	1530 (85.3)	<0.001
Condoms and methods of contraception	110 (10.2)	211 (11.1)	0.474	721 (70.5)	1126 (63.1)	<0.001

**Topics related to character and emotions**						
How to know when I will be ready to start dating	224 (20.6)	671 (34.9)	<0.001	762 (74.5)	1436 (80.3)	0.002
How to know when I will be ready to have sex	110 (10.2)	254 (13.4)	0.022	673 (65.5)	991 (55.7)	<0.001
What "falling in love" means	278 (25.7)	648 (33.9)	<0.001	872 (85.2)	1627 (89.8)	0.001
How to continue going out with a person without having sexual relationships	181 (16.8)	509 (26.7)	<0.001	746 (72.6)	1420 (79.6)	<0.001
How to know if the person I am dating is the right one to share my future life with	281 (26.0)	702 (36.8)	<0.001	839 (81.4)	1583 (87.8)	<0.001
How to better manage my feelings and emotions	363 (33.5)	883 (46.1)	<0.001	897 (86.9)	1714 (94.4)	<0.001
How to better manage my sexual drive/passion	133 (12.3)	291 (15.4)	0.036	741 (72.2)	1170 (65.9)	0.002
How to tell the difference between desire, sexual attraction and love	185 (17.1)	469 (24.7)	<0.001	859 (83.0)	1571 (86.6)	0.023

* Respondents marking the 4^th ^or the 5^th ^answer option in the question "I have talked to my parents about ...", with a five-point Likert scale labeled from a low "Nothing" to a high "A lot" score.

^† ^Respondents saying they would like to know more about each topic.

^‡ ^Proportions are calculated using denominators that range from 961 to 1085 (males) and from 1597 to 1937 (females) because some items were not responded by some participants.

^§ ^p value of the adjusted Wald test taking into account the clustered sampling scheme.

On most topics (biological as well as affective ones), more girls than boys reported conversations with their parents. The highest difference was found on the topic "girls' physical changes", with 9.0% of boys and 59.9% of girls indicating they talked "somewhat" or "a lot" about this topic with their parents (p value < 0.001). On the contrary, "boys' physical changes" was the only topic on which more boys than girls reported conversations with their parents (18.8% of boys and 10.7% of girls, p value < 0.001).

Survey participants were also asked whether they would like to know more about sexuality topics. A wide majority of participants said they would like to know more about all the contents presented. However, both boys and girls expressed greater interest in issues such as how to better manage feelings and emotions (86.9% boys, 94.8% girls) and what "falling in love" means (83.3% and 89.9%). On most topics, girls showed a higher desire to know more, except boys' physical changes, contraception, how to know when one is ready to have sex and how to better manage sexual drive: on these topics, boys' desire to know more was higher (Table 2).

We identified some problems regarding the youth's knowledge about the prevention of STIs and unintended pregnancies. When asked about the risk they believe may occur if one has sex with condoms, the percentages of respondents answering "none" or "I don't know" were 42.9% for risk of AIDS infection, 43.7% for risk of genital warts infection and 40.6% for pregnancy, with higher rates among boys (p = 0.007, p = 0.016 and p < 0.001 respectively) (data not shown).

Attitudes toward sexism were explored by asking the youth whether they agree with media using women or men as "sexual objects", or associating femininity or masculinity to having more sexual relationships. On both items, more girls compared to boys were significantly sensitive and disapproving of sexuality being misused in advertisements (Table 3).

**Table 3:** Opinions on sexism

Opinions	Male (N = 1096)	Female (N = 1949)	
	n * (%)	n * (%)	p^†^
I do not like it when the media (TV, ads, magazines, films...) show:			
- women as "sexual objects"; only giving importance to her body	435 (39.7)	1236 (63.4)	< 0.001
- men as "sexual objects"; only giving importance to his body	437 (39.9)	1099 (56.4)	< 0.001

I do not like it when the media (TV, ads, magazines, films ...) associate:			
- femininity to having more sexual relationships	378 (34.5)	1115 (57.2)	< 0.001
- masculinity to having more sexual relationships	378 (34.5)	1030 (52.8)	< 0.001

* Number of respondents marking the 4^th ^or the 5^th ^answer option in a five-point Likert scale labeled from a low "strongly disagree" to a high "strongly agree" score.

^† ^p value of the adjusted Wald test taking into account the clustered sampling scheme. Taking into account the 5 answer levels, the test for trend was also significant (p < 0.001) in the four variables.

After adjusting for sex, age and whether institutions were public or private, the students that believe condoms are 100% effective against AIDS, STIs and pregnancies were more likely to be sexually experienced (OR= 1.59; 95% CI 1.09–2.33). Students that are approving of pornography and masculinity and femininity being equated to having more sexual encounters, were as well more likely to be sexually experienced after the adjustments mentioned above (OR= 1.69; 95% CI 1.25–2.29).

## Discussion

The respondents of the study were representative of private and public schools of the Philippines. We performed weighted analyses in the descriptive results in order for them to be representative of Filipino students.

According to the Philippine National Statistics Office, 81% of Filipinos are Catholic, and 8.2% belong to other Christian religions [41], which is similar to our weighted sample distribution. Regarding the distribution of sex, institutions report higher enrollment ratios for girls than for boys. Specifically, in secondary education, net enrollment ratios (NER) are 54% for boys and 65% for girls [42]. Since sex ratio (male/female) for these ages is approximately 1 [43], this means that approximately 55% of students are girls in high schools. This accounts for the higher female presence in our sample.

Referring to our paper sample of teens, the main information source about love and sexuality is friends. This is similar to studies from Sweden, USA, United Kingdom, Czech Republic and Spain [44-49]. Existing literature likewise provides evidence that media (Internet, magazines) are the second source of information, outranking parents, as happens in our male sample [45,49]. A study in Nigeria, however, sets parents in the first place among in-school girls [50].

Literature shows that communication with parents protects against early sexual initiation and against risky behaviors [51,52]. Conversely, information sources which are mostly used in our sample (peers, media) are not usually described as ideal for educating teens [46,53]. At the same time, parents' opinion regarding sexuality and other related topics is well valued by teens in our study. This is confirmed by surveys which also show parents being rated as preferred sources rather than as actual sources [47]. Furthermore, parents' attitudes toward certain risk behaviors (such as smoking and drinking alcohol) seem to be protective against those behaviors in their children [54]. This seems to show that parents' opinions are indeed taken into account when given to children. There is therefore room for further encouraging parents to talk more with their children about sexuality, including aspects related to feelings and emotions that could help them make better sexual and reproductive choices. This is specially valid for daughters, who give in our data much importance to their parents' opinion.

With regard to knowledge of sexuality, we observe that teens in most cases (specially among girls) have not talked about sexuality topics with their parents, but that they would want to know more. We must also stress that teens' desire for information is not limited to the biological aspects of sexuality. In fact, they are much interested to know more about the emotional aspects of relationships and sexuality. Examples are to know more about how to manage one's feelings and sexual drive; meaning of "falling in love"; how to know if the person one is dating is the right person; and how to tell the difference between desire, sexual attraction and love. Having a better understanding of these issues can be very useful to avoid premature sex [52], and parents agree that these aspects should be addressed [55]. Indeed, these issues are related to the perceived well-being of teens. With sex education programs concentrating on biological information [36], they are in effect highlighting topics that are of relatively lower interest to teens while downplaying education in the affective aspects of human sexuality which could be a powerful means to empower teens to make healthier life choices [56]. To our knowledge, the issue of making emphasis on affective aspects is seldom brought up in sex education policies.

Regarding sex differences in this issue, we find that, in general, girls talk more with their parents about most topics, and also want to know more. Boys only talk more about their own physical changes, and have a bigger desire to know more about these changes and about topics that might be related to their higher sexual drive.

The teens of our study also have incomplete information on some biological facts related to sexuality. Concerning condom effectiveness, for example, several studies show that condoms are "risk reduction" measures with respect to unintended pregnancies, HIV infection and other STIs and should not, therefore, be presented as "risk avoidance" measures [57-61]. We find that around 40% of respondents (even more among boys) have the wrong belief that condoms are 100% effective or report not knowing their effectiveness. This overconfidence or lack of information can lead teens to underestimate the risks they are taking [62]. Teens who believe condoms can avoid rather than reduce the risk of STIs, underestimate the benefits of abstinence and mutual monogamy, as found in previous studies [37,63,64]; this perspective may negatively affect their decision-making in sexuality. Risk compensation may come into play and increase their vulnerability to infections and unintended pregnancies [65]. Briefly, this hypothesis suggests that the introduction of new technological approaches or messages of prevention could reduce the perception of risk at the broader population level and thus worsen the compliance with other basic preventive behaviors. In the end, higher risk-taking could offset the protective benefits theoretically associated to the new approach. For example, risk compensation was described as an explanation for the initial failure of seat belt laws to prevent road accident deaths because drivers presumed that wearing a seat belt would protect them from riskier driving [66,67]. More recently, other researchers have extended the concept of risk compensation to HIV prevention [68,69]. Campaigns mainly focusing on condom use at the population level could paradoxically lead to an increase in risky behaviors (such as the number of sexual partners), if the population perceives condoms to be absolutely safe, irrespective of specific sexual behaviors. As suggested by a recent community trial in Uganda, the overall effect of some interventions could be offset by riskier behaviors at the population level and thus hinder the targeted decrease of HIV incidence [70]. Our results are consistent with this cited paradoxical effect since the teens that falsely perceived condoms as being 100% effective were indeed more frequently sexually experienced. More might have to be done to improve the content and quality of the information conveyed to teens. While it seems important to give comprehensive information about all preventive measures, programs should be abstinence centered when targeting teens [71,72]. Teens should be clear that it is better to avoid rather than to reduce risks and they should be helped to achieve risk avoidance as it is indeed the only option 100% effective. By focusing on abstinence one can better avoid the slippery slope of risk compensation [62].

It is true that some studies about abstinence programs have found no statistically significant effects on sexual behavior [73-75]. However, some of these studies had several methodological problems, as reviewers themselves recognize, which might account for the lack of significant findings. Furthermore, other studies do find some abstinence encouraging programs being effective in both developed and developing countries [76-78]. Besides, even if lack of effects was proven, it should not be a surprise that a few hours of sex education programs in school are unable to compensate for the opposite message often conveyed by some parents, media, authorities and society in general [79]. The question is not whether to promote abstinence among teens, but rather how to achieve this.

Finally, the existing literature shows several dangers in the generalization of sexism. The American Psychological Association points out several problems associated to the sexualization of girls [80]. These include cognitive difficulties, mental health problems and risk behaviors. On the other hand, boys' exposure to pornography increases the risk of aggressiveness, rape myths and gender stereotypes, all of which may be indirectly harmful for women and equality between males and females [81-83]. In our sample, we observe that while sexism is rejected by a majority of girls, it is accepted by most boys. Most males do not seem to find anything wrong with the misuse of men or women as sexual objects, or associating masculinity or femininity to having more sexual relationships. Having these aforementioned opinions and perceptions was likewise associated with a higher incidence of sexual experience in our study.

There are several limitations in our study. First of all, the cross-sectional nature of any study does not enable to easily infer causality between dependent and independent variables. However, some insight is possible to understand the teens' feelings and opinions, and how these dispositions consequently affect their behavior. Cross-sectional studies do have the advantage of being less costly and thus more efficient to obtain certain useful results. Our data do suggest sensible and plausible associations. For example, the association between perceptions and beliefs about condom effectiveness, sexism and sexual experience are consistent with the theory of risk compensation as described by other researchers [69]. In addition, reverse causation, i.e. that early sexual initiation produces incorrect knowledge about condom effectiveness, does not seem very plausible. The fact that more boys than girls want to know more about controlling their sexual drive and more girls than boys want to know more about how to manage their feelings is consistent with the natural mindset of each sex and what is expected. Aforementioned socio-demographic data are likewise consistent with existing population data for the Philippines. In summary, we did not find inconsistent responses nor important alternative explanations of our findings.

Another possible limitation is that our data is based on self-reported responses. It is notable, however, that our results are not what one would expect from respondents giving socially desirable answers. Research indicates that self-reported data such as those found in Youth Risk Behavior Surveys (YRBS) of the United States can be gathered credibly from youth surveys [84]. Internal reliability checks were used to identify the percentage of students who possibly falsify their answers. To obtain truthful answers, students were made to understand why the survey is important, and procedures were developed to protect their privacy and allow for anonymous participation.

The survey environment, questionnaire design and content, edit checks, logic within groups of questions, and some comparisons of our results with other studies give us confidence on the validity of our data.

As described in the methods section, the in-class and casual setting where the questionnaires were administered, has presumably minimized invalid responses because respondent privacy and anonymity were ensured. Furthermore, students were adequately instructed to leave any discomforting question blank. Students sat as far apart as possible throughout the survey venue and had an envelope to cover their responses. Only a few skip patterns were used in the questionnaire and, in any case, they were used in such a way that the difference in the time needed to complete the questionnaire between youth with or without sexual experience was insignificant. The questionnaire was designed to suit the reading level of at least a junior high school student.

The questionnaire was previously piloted on a sample of 180 students in order to assure not only comprehension and cultural relevance of items, but also to avoid leading questions that may influence students' responses. In summary, we have no reason to believe that self-reporting could have compromised our results.

Despite its limitations, our study has several strengths. The analyses we have performed and presented are consistent with our sample being representative of the Filipino student youth. To our knowledge, this is the first representative study of a student population in the Philippines that has studied the issues of relationships, love and sexuality in such depth. Since STIs are increasing all over the world and STIs are associated to having more lifelong sexual partners, and the latter with earlier sexual initiation, the study of whether certain messages are associated to earlier sexual initiation is relevant across different cultures and countries. There are no studies associating the perception of 100% condom effectiveness with earlier sexual debut. This is the most novel aspect of our paper, and it is also the aspect presented with multivariate adjustment. Our data bring up the important issue that teens themselves are requesting more emphasis on affective aspects of human sexuality when educating them. Furthermore, beyond the issue of external validity due to the representative nature of our sample, its large sample size has enabled us to perform better statistical adjustment where needed, analyses accounting for the clustered sampling strategy and thus improve the validity of our results.

## Conclusion

Having a better understanding of what teens feel and think about relationships, love and sexuality, seems to be an important consideration in planning public health strategies to address common reproductive health problems in teen populations. This study highlights that Filipino students do not communicate as much as they would want with their parents on these issues. It seems that more can be done to improve parent-child communication as friends and the Internet are not the best information channels. Aside from improving the information source, more has to be done also to improve the content and quality of the information conveyed to teens. True informed choice and empowerment goes hand by hand with accurate information. In particular, condoms should be presented for what they are: a risk reduction strategy and never for risk avoidance. Survey findings seriously suggest that some messages conveyed to teens can indeed be harmful as these are associated with earlier sexual initiation. More public health resources should be spent on the maintenance of the lifestyle that better protects youth, i.e., in the case of this study, a lifestyle that is truly risk avoiding and beneficial to a larger section of the targeted teens. Our data suggests teens are requesting help to achieve a healthier lifestyle, and they are in fact more interested in character education encompassing affective aspects of sexuality rather than biological information. Global strategies should seriously take this request into consideration.

## Competing interests

The authors declare that they have no competing interests.

## Authors' contributions

JI, CLB, VAB, FOG, MCC and ANT designed the study and the questionnaire. CLB, AO and JI analyzed the data. AO and JI made the first draft of the paper. All authors contributed to the final manuscript.

## Pre-publication history

The pre-publication history for this paper can be accessed here:

http://www.biomedcentral.com/1471-2458/9/282/prepub
